# Cold Formalin Fixation Guarantees DNA Integrity in Formalin Fixed Paraffin Embedded Tissues: Premises for a Better Quality of Diagnostic and Experimental Pathology With a Specific Impact on Breast Cancer

**DOI:** 10.3389/fonc.2020.00173

**Published:** 2020-02-19

**Authors:** Enrico Berrino, Laura Annaratone, Umberto Miglio, Elena Maldi, Chiara Piccinelli, Erica Peano, Davide Balmativola, Paola Cassoni, Alberto Pisacane, Ivana Sarotto, Tiziana Venesio, Anna Sapino, Caterina Marchiò

**Affiliations:** ^1^Department of Medical Sciences, University of Turin, Turin, Italy; ^2^Pathology Unit, Candiolo Cancer Institute, FPO-IRCCS, Candiolo, Italy; ^3^Pathology Unit, Città Della Salute e Della Scienza di Torino, Turin, Italy

**Keywords:** fixation, cold formalin, DNA fragmentation, molecular diagnostics, breast cancer, diagnostic accuracy, oncology, biomarkers

## Abstract

Formalin fixation and paraffin embedding (FFPE) represent the standard method to preserve tissue specimens for diagnostic pathology, however formalin fixation induces severe fragmentation of nucleic acids. We investigated whether formalin fixation at 4°C could preserve DNA integrity in FFPE specimens. Paired samples from 38 specimens were formalin fixed at room temperature (*stdFFPE*) and at 4°C (*coldFFPE*), respectively. Two independent cohorts were prospectively collected, cohort A (collected 6 years prior to the study, *n* = 21), cohort B (collected at time of the study, *n* = 17). DNA was extracted and its integrity evaluated with a qPCR-based assay that produces a normalized integrity index, the QC score (ratio between the quantity of a long and a short amplicon of the same gene). We observed higher QC scores in *coldFFPE* compared to *stdFFPE* samples (mean values: 0.69 vs. 0.36, *p* < 0.0001) and *stdFFPE* breast cancer specimens showed the most detrimental effect overall. Comparable QC scores were obtained between *coldFFPE* tissues of both cohorts; conversely, DNA integrity of *stdFFPE* was significantly lower in cohort A compared to cohort B (*p* < 0.0001). Of note, QC scores of *stdFFPE* (but not of *coldFFPE*) samples were significantly reduced following 6 months of storage (*p* = 0.0001). Monitored formalin fixation at 4°C outperforms standard fixation in ensuring high-quality DNA, which is key to feasibility of downstream high-throughput molecular analyses. An important effect was observed over storage time, thus suggesting a likely better preservation of archival samples when this cold fixation protocol is used.

## Introduction

The harmonization of pre-analytic procedures represents the corner stone of optimal diagnosis in pathology (1, 2). The preanalytical phase in pathology includes different steps, spanning from transportation of tissue specimens from surgical theaters to pathology laboratories, grossing, and fixation of tissues (type of fixative and duration of fixation are key features in this respect). Tissue fixation in formalin with generation of formalin-fixed paraffin embedded (FFPE) tissues blocks represents the standard method for tissue specimen processing and archival in diagnostic pathology at present. Of note, heterogeneous tissue handling methods typically lead to inefficiency and poor reproducibility in pathology laboratories. Tissue fixation in formalin, with the generation of formalin-fixed paraffin embedded (FFPE) tissue blocks, represents the standard method for tissue specimen processing and archival in diagnostic pathology. With the tremendous advances of precision medicine pathologists face the challenge to integrate morphology and immunophenotyping with genetic and epigenetic analyses, which require purification of good quality nucleic acids from FFPE blocks. In addition, FFPE tissue blocks preserved in pathology archives may constitute the substrate for comprehensive “omics” strategies, in the context of both prospective and retrospective experimental studies.

It is widely accepted that formalin fixation exerts a blasting effect on both DNA and RNA, with damages comprising fragmentation, non-canonical cross-linkage and base alterations, with critical proportional consequences related with storage time (3, 4). Of note, Polymerase-Chain Reaction (PCR)-based next generation sequencing (NGS) methods are strongly influenced by formalin artifacts, reducing the library performance. The extensive fragmentation of DNA purified from FFPE samples usually leads to lower coverage of unique reads in whole genome and whole exome sequencing approaches (5), but it may also decrease the success rate of amplicon-based methods due to reduced size of DNA templates (6). Moreover, the low quality of DNA from FFPE samples also stems from formalin induced base artifacts within the sequences, generating false mutation calls, in particular in sub-clonal experiments (7).

We have previously reported on a protocol, i.e., cold formalin fixation, which improves RNA quality without interfering with the formalin ability to preserve both tissue structure and antigen reliability for immunohistochemical analyses (2). This modified formalin fixation may represent an easy alternative to conventional formalin fixation ensuring a better preservation of analytes. In addition, a standard method is needed to identify those DNA samples reaching the minimal parameter to obtain robust results. For the large majority of genomic downstream applications, the assessment of DNA size distribution, the ratio between long and short fragments as well as the amplifiability of samples can contribute as parameters to define a quality score (8).

Based on these premises, the aim of our study was to comprehensively characterize a series of DNA samples purified from tissues derived from the same surgical resection but processed with two different fixation protocols (standard fixation vs. cold fixation at 4°C), and to assess whether results would differ based on the tissue of origin. Fluorometric, spectrophotometric and qPCR-based methods were applied to assess DNA integrity.

## Materials and Methods

### Sample Collection and Sampling Procedures

The 38 paired samples included in the study were *ad hoc* collected from surgical specimens handled by under-vacuum packing and cooling (VPAC), as previously described (2, 9, 10). All of the surgical specimens enrolled in this study were at least 2 cm in size to allow for proper parallel sampling of comparable size and thickness. Informed consent was obtained from all individual participants included in the study (protocol “Profiling” 001-IRCC-00IIS-10, approved by the Ethical Committee of Fondazione Piemontese per l'Oncologia- Istituto di Ricerca e Cura a Carattere Scientifico of Candiolo). All experimental procedures were performed in accordance with relevant guidelines and regulations. It should be noted, however, that the sampling did not affect the diagnostic process as the adopted procedure did not require additional samplings, rather included a variation of tissue processing for parallel samples, already validated in a previous study as non-interfering with morphological evaluation (2). Of note, all of the tissue samples were available to the pathologist in charge of signing out the final diagnosis. Each tumoral lesion was sampled in parallel as follows: (i) *standard fixation procedure (stdFPPE):* samples were fixed for 24 h in 4% neutral-buffered formalin (NBF) at room temperature (RT). Subsequently, samples were processed to paraffin embedding with an automatic tissue processor and embedded in paraffin wax; (ii) *cold fixation procedure (coldFFPE):* the sample was immersed in pre-cooled 4% NBF and fixed at 4°C for 24 h. For this protocol, specimens were dehydrated in ethanol 95% at 4°C for 4 h and then followed the same *stdFFPE* sample processing without the first ethanol 95% step by using the automated tissue processor. All of the methods were performed in accordance with the relevant guidelines and regulations.

**Figure 1:**
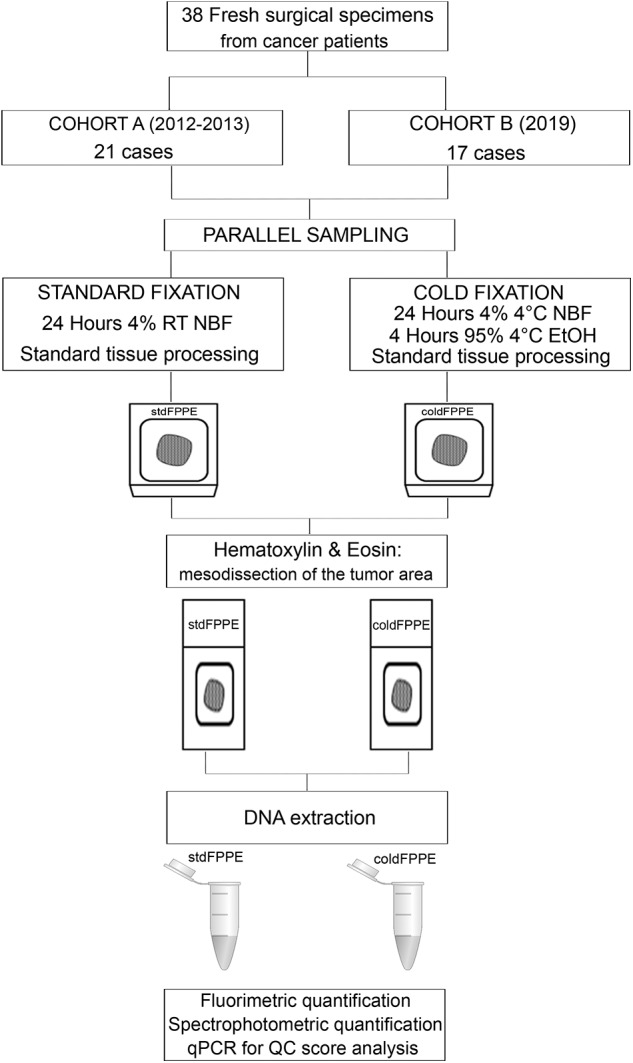
Design of the study. The full cohort comprised 38 cancer specimens that were collected and sampled in parallel to allow standard fixation (i.e., at room temperature) and cold formalin fixation (i.e., at 4°C). Following processing and tissue sectioning the H&E slides were reviewed to identify the tumor area that was mesodissected for DNA extraction. Two independent cohorts were prospectively collected: Cohort A, whose sampled were collected 6 years prior to the study and DNA extraction performed at present time; Cohort B, whose samples were collected at time of the present study with contextual DNA extraction. For 14 samples from Cohort B, i.e., corresponding to those samples for which at least 6 months elapsed from collection, two DNA extractions were performed: at baseline (at time of collection/fixation) and after 6 months of storage/archival. On the total 90 DNA samples we performed fluorometric and spectrophotometric quantifications and we ran a qPCR with the DEPArray™ FFPE QC Kit. RT, room temperature; NBF, neutral buffered formalin; EtOH, ethanol.

Out of the 38 cases, 21 pairs were collected and fixed 6 years prior to the present study (Cohort A, collected between 2012 and 2013) and 17 samples prospectively collected at time of the present study (Cohort B, collected in early 2019) (Figure 1). Cohort A included 11 breast carcinomas of no special type, three colorectal adenocarcinomas, three cases of lung adenocarcinoma, two gastric adenocarcinomas, and two thyroid follicular carcinomas (Supplementary Table 1). Cohort B was composed of eight cases of colorectal adenocarcinoma, two Gastro-Intestinal Stromal Tumors (GISTs), two lung adenocarcinomas, one intrahepatic cholangiocarcinoma, one splenic metastasis of endometrial carcinoma, one high grade serous-papillary ovarian carcinoma, one case of pleomorphic undifferentiated sarcoma and one adenocarcinoma of the gallbladder (Supplementary Table 1).

### Nucleic Acid Extraction and Quantification

A pathologist evaluated histological and pathological features of the cases included in both cohorts and the tumor area was identified on the hematoxylin and eosin (H&E) stained slide before proceeding to the experimental procedure for both *stdFFPE* and *coldFFPE* samples. Data were analyzed anonymously. Five 8-μm thick sections were dissected and DNA samples were purified from all the 38 FFPE pairs for a total of 72 DNA samples. In addition, in 14 cases from cohort B for which at least 6 months elapsed from collection paired *stdFFPE* and *coldFFPE* samples underwent a second DNA extraction 6 months after collection/fixation.

DNA was extracted using the QIAamp DNA FFPE Tissue Kit (Qiagen, Hilden, Germany) according to the manufacturer's protocol, following an overnight 56°C tissues lyses allowing a complete tissue digestion without fragmenting the DNA. DNA was eluted in 40 μL of nuclease-free water and quantified using the Qubit 3.0 Fluorimeter (Life Technologies, Wilmington, DE, USA) following the protocol of High Sensitivity DNA Kit (Life Technologies, Eugene, OR, USA). To check for any possible contaminant on the purified samples, DNA concentration was also evaluated using the DeNovix DS-11 UV-Vis Spectrophotometer (DeNovix, Wilmington, DL, USA) to obtain the 260/230 and the 260/280 nm ratios.

### DNA Fragmentation Analyses

DNA fragmentation was evaluated by using the DEPArray™ FFPE QC Kit (Menarini-Silicon Biosystem, Bologna, Italy). This qPCR-based kit is a multiplex reaction composed of two primers pairs flanking two regions of 54 and 132 bp of the same genomic locus. To quantify the amount of the produced amplicons, a standard curve for each primer pair was generated. To infer the DNA integrity level, the ratio between the quantity of the long amplicon (e.g., most conserved DNA) and the short amplicon is calculated. This ratio, named QC score, is a normalized number tending either to 1, in a context of highly conserved DNA, or to 0, in a scenario of diffuse DNA degradation.

Briefly, the standard curve points were produced with serial 1:10 dilutions from an undiluted standard with a known concentration of 20,000 pg/μL. To perform the assay, 8 μL of DNA with a concentration of 2 ng/ μL were added in two mix composed by 10 μL of the Master Mix and 2 μL of the Short or Long Primer mix for a final volume of 20 μL. The thermal cycle was run on a RotorGene Q instrument (Qiagen) and contemplated an initial step of DNA denaturation for 10 min at 95°C followed by 40 cycles of 20 s at 95°C denaturation and 1 min at 57.5°C annealing/extension with the detection of FAM fluorescence signal at 520 nM. To obtain optimal results, slope and *R*^2^ parameters of the standard curve may be between 3.1 and 3.6 and 0.99, respectively. The fluorescence unit threshold was set at 0.04 for all the experiment.

All samples and standards were analyzed with technical triplicates in each experiment. The amount of amplified DNA was checked analyzing the quantity of the short and long amplicons assay products. By comparing the average Ct of the three replicates with the standard curve the relative quantification (RQ) of PCR product was obtained using the formula 10^(CTsample-INTERCETstdcurve/SLOPEstdcurve)^ for both the short and long amplicon. The QC score was obtained by dividing the long amplicon RQ by the short amplicon RQ.

### Statistical Analyses

Statistical analyses were carried out using GraphPad Prism statistical software v8.0 (GraphPad Software, La Jolla California USA). After normality test, we applied both the unpaired and paired distribution test to evaluate significant differences in QC scores between the two-fixation methods. The unpaired distribution test was used to assess differences by considering the QC scores as independent measurement of uncorrelated samples, whereas the paired distribution test evaluated the statistical distribution considering the different fixation of the same sample as repeated and correlated data. The Pearson's correlation coefficient test (r) was used to determine the correlation between QC scores of corresponding *stdFFPE* and *coldFFPE* samples to determine any intra-specimen influences.

We first analyzed the total cohort by merging the data obtained from the two sets and then by separately considering cohort A and cohort B. *p*-values of <0.05, with 95% confidence intervals (CI), were considered as statistically significant.

## Results

### Cases and Tissue Morphology

The H&E sections of corresponding *stdFPPE* and *coldFFPE* samples were evaluated by pathologists blinded to the experimental procedures of fixation. Only tumor cell areas were selected for dissection and DNA purification. Tumor cell composition was comparable between corresponding samples for each case; minor differences were observed in terms of relative percentage of immune cells populating the intertumoral stroma. Necrotic areas were carefully avoided through mesodissection. Only two sample pairs were characterized by large necrotic areas interspersed among tumor cell clusters and most likely contained a non-negligible degree of necrosis in the extracted material, nevertheless no evident technical-derived alterations were detected. Of note, the pathologists involved in the assessment of morphological features did not raise observations on fixation artifacts in any of the samples.

### DNA Quantity and Absorbance Quality

To determine any influence of the different fixation protocol on DNA quantity and quality, we both applied fluorometric and spectrophotometric methods to evaluate the DNA amount. DNA concentration and ratios are reported in Supplementary Tables 1, 2.

From the standard fixed samples, we purified a mean level of 4.2 (range: 0.2–15.6 μg) and 16.2 μg (1.9–57.7 μg) of DNA analyzed with Qubit or DeNovix instruments, respectively. No significant differences (*p* = 0.2) were detected by comparing these data with the DNA quantity obtained from *coldFFPE* samples, which were slightly higher by the fluorometric (mean: 4.9 μg, range: 0.3–16.4 μg) and spectrophotometric measurement (mean: 20.37 μg, range: 2.4–55.8 μg).

**Figure 2:**
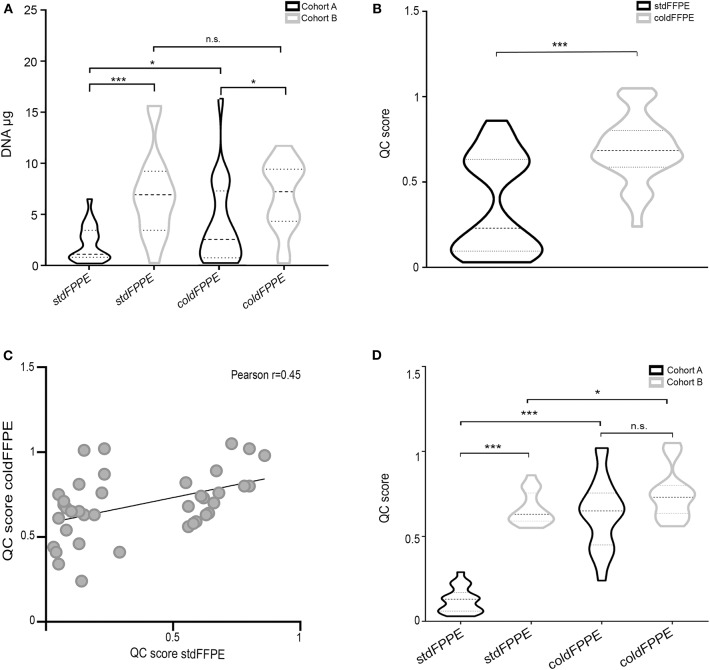
Quantity and quality of DNA extracted from parallel samples. **(A)** DNA quantification of samples. Violin plots representing the DNA μg purified from the specimens, grouped according to the fixation methods (*stdFFPE* and *coldFFPE*) and the time of cohort collection [Cohort A (collected 6 years prior to the study with DNA extraction at present time); Cohort B (collected at time of the present study with contextual DNA extraction)]. Cohort A was characterized by higher DNA yields for *coldFFPE* compared to *stdFFPE* samples (4.1 μg vs. 2.1 μg, *p* = 0.04). As for Cohort B, violin plots showed a median-around density distribution for both *stdFFPE* and *coldFFPE* samples that was significantly higher compared the same fixation protocol of Cohort A (*p* = 0.03 for cold fixation and *p* =0.0001 for standard fixation). **(B)** Violin plots representing the QC score distribution in the groups clustered according to fixation method. The *stdFFPE* cohort was characterized by heterogeneous QC scores with several samples in the low range (wider diameters of the black violin) and the DNA fragmentation was statistically significantly higher than in *coldFFPE* (gray violin). **(C)** Dot plot illustrating the Pearson correlation between the QC scores of corresponding samples fixed with the two protocols. The absence of a robust correlation suggests the reduced influence of the intra-individual fragmentation on the QC score. **(D)** QC distribution among the sample sets. Violin plots representing the QC score of the samples, which are grouped according to the fixation methods (*stdFFPE* and *coldFFPE*) and the time of collection [Cohort A (collected 6 years prior to the study with DNA extraction at present time); Cohort B (collected at time of the present study with contextual DNA extraction)]. The QC scores of *coldFFPE* samples are comparable between the two cohorts, regardless of time of collection/fixation (*p* = 0.79), whereas the QC score of *stdFPPE* samples are significantly lower in Cohort A compared to Cohort B (*p* < 0.0001). Notably, the DNA purified from Cohort B samples displayed a better preservation in both *stdFFPE* and *coldFFPE*, nevertheless *coldFFPE* samples still showed a higher QC distribution. n.s. not significant, *p < 0.05, ***p < 0.001.

When considering Cohort A only, a significantly higher DNA quantity was detected for *coldFFPE* compared with *stdFFPE* samples (mean values: 4.1 μg vs. 2.1 μg, *p* = 0.04) using the Qubit fluorometer instrument, which was not appreciated in Cohort B. For both fixation methods the nucleic acid yield was significantly higher in Cohort B than in Cohort A (*p* = 0.03 and *p* < 0.0001 for *coldFFPE* and *stdFFPE* samples, respectively), however the difference was less evident when comparing *coldFFPE* than *stdFFPE* specimens (40% of reduction against 70%) (Figure 2A).

No correlation between DNA amount and type of tumors was detected. Finally, the fixation protocol did not influence the DNA quality, defined as the absorbance ratio between 230/260 and 260/280 nm, which resulted comparable between the pairs.

### DNA Fragmentation Analysis

The qPCR-based DEPArray™ FFPE QC Kit returned a QC score for each sample, a normalized evaluation of DNA amplifiability. From a technical standpoint, the mean *R*^2^ scores of the standard curves were 0.994 and 0.993 for the short and the long amplicon, respectively, and the line slopes were comprised in the optimal range defined by the manufacturer's protocol.

All DNA samples were successfully amplified, and a QC score was generated. More in detail, the *stdFFPE* DNA samples were characterized by mean QC score of 0.36 (range: 0.03–0.86), which was significantly lower than the corresponding value in *coldFFPE* samples (mean: 0.69, range: 0.34–1.05). By comparing the QC score distributions of the two sets of samples, both the paired and unpaired tests reached the statistical significance (*p* < 0.0001) (Figure 2B). We wondered whether the intrinsic fragmentation of the samples could have influenced this difference. In this context, the Pearson correlation test described an independent trend for the QC value for the two different fixation methodologies in the same sample (*r* = 0.45) (Figure 2C).

When samples were clustered according to the time of fixation (Cohort A vs. Cohort B) we observed that the QC scores of *coldFFPE* samples were substantially comparable between the two cohorts, regardless of time of fixation (*p* = 0.79), whereas the QC score of *stdFFPE* samples was significantly lower in Cohort A compared to the more recent prospectively accrued samples of Cohort B (*p* < 0.0001) (Figure 2D). Within Cohort A, *stdFPFPE* samples harbored the highest level of DNA fragmentation overall, with a mean QC level of 0.12 (range: 0.03–0.27), in contrast with the corresponding *coldFFPE* samples that showed a 6-fold increase in the QC average (mean QC: 0.63, range: 0.24–1.02; *p* < 0.0001). On the other hand, the DNA purified from the 17 paired samples of Cohort B displayed a relatively better preservation in both *stdFFPE* and *coldFFPE*, nevertheless *coldFFPE* samples still showed a statistically significantly higher QC distribution (*p* = 0.0016 and 0.038, paired and unpaired *t*-tests respectively, Figure 2D).

### DNA Integrity and Site of Origin of the Tumor

**Figure 3:**
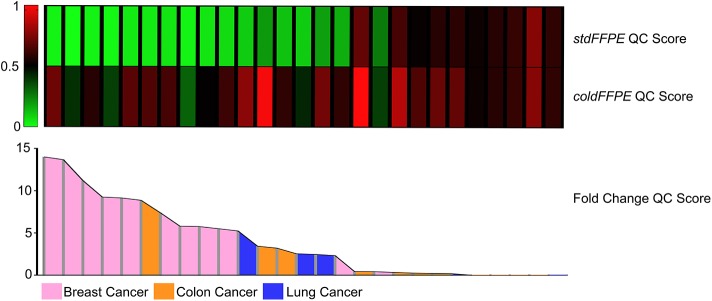
QC score and QC score fold change in breast, colon, and lung carcinoma samples. The heatmap represents the QC score for purified DNA from *stdFFPE* and *coldFFPE* samples. The histogram shows the fold changes of the QC score in *coldFFPE* compared to the corresponding *stdFFPE* samples. The order of the samples in the heatmap is defined by the color in the histogram. All the breast *stdFFPE* samples shows the lowest QC score level, which is less heterogenous among the *coldFFPE* specimens. Nine of the first ten samples with and increased level of DNA integrity have a mammary site of origin, confirming the specific improvement for breast cancer tissue.

We checked any possible association between the site of origin of the tumor and the DNA preservation efficiency of the two processing methods. The heterogeneity of our population allowed to reach enough numerosity to obtain statistically informative results only for breast, colorectal, and lung carcinomas. We observed that the DNA purified from breast carcinomas showed higher degrees of fragmentation compared to colorectal and lung adenocarcinomas (Figure 3). We obtained the lowest QC scores from *stdFFPE* breast carcinoma samples, and these values were significantly lower compared to all the other samples, either *stdFFPE* or *coldFFPE* (*p* = 0.0009). By focusing on the same samples treated with cold formalin, breast specimens confirmed the tissue-specific DNA degradation, compared with lung and colon cancer tissues (*p* = 0.02), more pronounced within the standard fixed samples (*p* = 0.0002). By investigating the intrasample QC score ratio between *stdFFPE* and *coldFFPE*, the highest advantage from cold formalin fixation was observed for DNAs purified from breast carcinoma specimens, with average 7.7-fold increase in the long amplicon in the *coldFFPE* compared to the *stdFFPE* samples (Figure 3).

### DNA Integrity Following 6 Months of Storage

**Figure 4:**
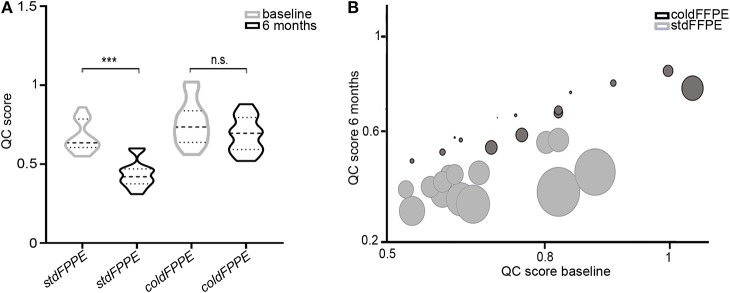
QC score distribution in the subgroup analysis of the 14 tissue pairs with a second DNA extraction after 6 months from collection/fixation. **(A)** Violin plots representing the QC scores in a head to head comparison between DNA extraction at baseline and after 6 months, subdivided by standard fixation (*stdFFPE*) and cold fixation (*coldFFPE*). Significantly lower QC scores are observed after 6 months in *stdFFPE* samples, whereas comparable QC scores are displayed for *coldFFPE* samples. **(B)** Bubble plot of the QC score for each patient. The plot showed the basal QC score on the Y axes and the 6-month QC score on the X axes, paired for each sample. The bubble size illustrates the drop in terms of QC score for each couple of DNA samples purified after 6 months of archival. n.s. not significant, ***p < 0.001.

Based on the lowest level of DNA integrity observed in *stdFFPE* samples of Cohort A and on the better values of QC scores detected in *stdFFPE* samples of Cohort B, we hypothesized that the time of storage could have had an impact on DNA integrity. In subgroup analysis of the 14 tissue pairs that underwent a second extraction after 6 months from collection and fixation we were able to perform a head to head comparison of the QC score values of DNA extracted following FFPE storage of 6 months to those of the DNA extracted at time of collection (Supplementary Table 2). We observed significantly lower QC scores for the *stdFFPE* samples (*p* < 0.0001; with a mean percentage loss of 35%) and comparable QC scores for the *coldFFPE* samples (*p* = 0.131, with a mean percentage loss of 9%) (Figure 4).

## Discussion

Our experimental study shows that formalin fixation of tissue specimens at 4°C (cold fixation) leads to a better preservation of DNA as exemplified by the significantly lower levels of fragmentation in DNA samples obtained from *coldFFPE* as compared to those from *stdFFPE* tissue samples. In addition, the results in our hands suggest an important impact of time of storage of FFPE samples on DNA integrity that can be circumvented by cold fixation. This seems to be particularly important for breast cancer specimens, which showed the most detrimental effect with standard fixation overall.

It is universally acknowledged that optimal DNA quality is obtained from fresh frozen tissue specimens, which represents also the backbone of tissue biobanking. Nevertheless, systematic freezing in pathology laboratories is seldom feasible on a routine basis, being restricted mainly to research Institutes where a proper Biobank is in place with dedicated personnel. On the other side we should take into account that diagnostic pathology is constantly reshaping due to the contribution of several molecular assays (including NGS-based approaches) to the diagnostic process, leading to either a better definition of the lesion, or a better prognostic and/or predictive stratification of the disease (precision medicine). For instance, depending on the site of origin and on the histologic type of a tumoral lesion genetic alterations such as mutations, translocations and gene expression signatures are currently being assayed in clinical practice [reviewed in (11)]. As pointed out by Schillaci et al. the primary goal of radiological and pathological evaluation is to increase the quality of life of oncological patients, both through the reduction and the invasiveness of the methods as well as by the accuracy of molecular analyses (12). Feasibility, robustness and reproducibility of molecular assays are therefore key in this respect and these features are strictly dependent on the integrity of DNA/RNA, which derives from a proper management of tissue during the preanalytical phase, including formalin fixation. Although several molecular assays have been designed and optimized on FFPE tissue samples and robustness has been shown, there are important scenarios in which genomic analyses on DNA extracted from FFPE samples are challenging. High throughput sequencing methods, such as Tumor Mutation Burden, somatic detection of *BRCA1* and *BRCA2* single nucleotide variants and copy number variations that can address patient to targeted therapeutic approaches (13), are strongly influenced by DNA integrity (14, 15). The pilot study for the 100,000 Genome Project excluded significant number of samples due to the poor quality of DNA extracted from FFPE samples, and the enrolled tissues revealed coverage unbalance after sequencing (16).

Cold formalin fixation has been used in other studies, mainly focused on RNA integrity. Our group (2) and others (17) have highlighted a better preservation of RNA molecules, whereas very little is known in terms of DNA integrity. Our systematic approach that analyzed the DNA fragment distribution in paired tissues with different formalin fixation methods provides direct evidence of a lesser degree of DNA fragmentation in *coldFFPE* samples, which showed significantly higher QC score values compared to *stdFFPE* DNA samples. Of note, the degree of statistically significant difference between *coldFFPE* and to *stdFFPE* DNA samples was higher in Cohort A, composed of specimens collected 6 years prior to the present study and whose DNA was recovered at time of the present study, compared to the Cohort B, composed of samples collected and extracted at present time. It is important to note that QC scores of the *coldFFPE* were comparable between the two cohorts, thus demonstrating consistency in obtaining high quality DNA from *coldFFPE* samples, whereas we observed relatively higher QC scores in *stdFFPE* DNA samples belonging to the prospective cohort, which rendered the difference with *coldFFPE* samples less evident, even though still statistically significant.

These data prompted us to assess whether an effect due to length of FFPE block storage could have contributed to the difference. This would be particularly relevant, as archival FFPE tissue blocks are a source of retrospective and prospective samples for translational research (18, 19) and, if a better method of preservation is identified, pathology archives may become an invaluable source of good quality DNA for future studies. Guyard et al. reported a systematic quantification of the time-dependent degradation of DNA in FFPE specimens and they detected a loss of both quantity and quality of DNA extracted from the same FFPE samples stored over a period of several years (4). In our study, we ran a subgroup analysis on 14 samples that underwent a second DNA extraction after 6 months from collection and fixation. We demonstrated a significant QC score loss in *stdFFPE* samples compared to the first extraction, whereas comparable results were obtained between first and second DNA extraction for *coldFFPE* samples, hence strongly suggesting a better preservation of DNA in tissue specimens fixed in cold formalin. As a possible mechanism contributing to this phenomenon one could hypothesize that cold formalin fixation may be able to reduce both the first enzymatic degradation of DNA by blocking the DNAse activity (20) and the formalin-dependent crosslinks that lead to DNA fragmentation (21).

Another important parameter derived from our data relates to the tissue type. We detected the highest advantage for DNA integrity in *coldFFPE* breast cancer specimens, as compared with *coldFFPE* specimens derived from colorectal, lung, and thyroid carcinoma samples. A tissue specificity for the DNA quantity and quality has been already reported by other groups. Bonin et al. showed variable yield and quality of nucleic acid extraction for different tissue (22) and Guyard et al. (4) reported a reduced DNA integrity in colorectal cancer FFPE tissues, probably associated with physiological characteristics, compared with lung, and urothelial tumors. The fat tissue the mammary gland is composed of may hamper an efficient formalin fixation, yet our results seem to suggest that breast cancer specimens may benefit form cold formalin fixation.

Our study has some limitations, mainly related to the limited sample size. Even though we were able to validate our results in two independently collected series of cases, we cannot rule out the possible association about tissue specificity for the less represented tumor types here included. Second, we could not systematically monitor the DNA fragmentation trend in all of the specimens over time. Nevertheless, the consistent data obtained in all the 14 sample pairs of the subgroup analysis we were able to perform strongly supports the contention of a better DNA preservation in cold fixed samples.

Finally, our analyses were carried out on parallel sampling of lesions from surgical samples only. We have preliminary data in our hands (unpublished results) demonstrating that cold fixation in core biopsies of breast carcinomas results in optimal morphology and reliable protein detection by immunohistochemistry-based assays. Further studies are warranted to ascertain whether the advantage in terms of DNA integrity demonstrated for FFPE samples of surgical specimens is also observed in core biopsy and cytology samples, which are smaller in size and may undergo a shorter duration of formalin fixation.

Despite these limitations, the precise quantification of the degree of DNA fragmentation that we performed with a normalized evaluation of the QC score revealed a clear benefit for DNA preservation in samples fixed in cold (4°C) formalin. Hence, we here provide a method to limit DNA degradation that is likely to (i) ensure a better performance of molecular diagnostic tests, (ii) enable a higher complexity of genomic analysis on DNA extracted from FFPE samples, (iii) anticipate a paradigm shift in pathology laboratories with the creation of FFPE archival samples that may be better preserved over time and foster therefore a higher accuracy and throughput of predictive molecular pathology assays, which have the potential to ultimately impact on the quality of life of breast cancer patients in the era of precision medicine.

## Data Availability Statement

The datasets generated for this study are available in the Supplementary Materials.

## Ethics Statement

The studies involving human participants were reviewed and approved Ethical Committee of Fondazione Piemontese per l'Oncologia- Istituto di Ricerca e Cura a Carattere Scientifico of Candiolo, CANDIOLO, Prot. Profiling 001-IRCC-00IIS-10. All subjects gave written informed consent in accordance with the Declaration of Helsinki.

## Author Contributions

CM and AS: conceptualization and funding acquisition. EB, LA, UM, CP, EP, EM, DB, PC, AP, IS, TV, and CM: methodology. EB, CM, and AS: formal analysis and investigation. EB and CM: writing—original draft preparation. CM, AS, LA, and TV: writing—review and editing.

### Conflict of Interest

CM has received personal/consultancy fees from Axiom Healthcare Strategies, Daiichi-Sankyo, MSD, Roche and Bayer, outside the scope of the submitted work. The remaining authors declare that the research was conducted in the absence of any commercial or financial relationships that could be construed as a potential conflict of interest. The handling editor declared a past co-authorship with one of the authors CM.
