# Supplementary material for: Cold Formalin Fixation Guarantees DNA Integrity in Formalin Fixed Paraffin Embedded Tissues: Premises for a Better Quality of Diagnostic and Experimental Pathology With a Specific Impact on Breast Cancer
Source: Front Oncol. 2020 Feb 19;10:173. doi: 10.3389/fonc.2020.00173 (PMC7042205; doi:10.3389/fonc.2020.00173)
Supplement: Supplementary file 1 [file Table_1.DOCX]

**Supplementary Table 1: Details on DNA quantity and quality for each pair of the whole series of cases included in the study.**

coldFFPE: cold formalin fixed paraffin embedded sample fixed; stdFFPE: standard (room temperature) formalin fixed paraffin embedded; µg Spectrophotometer: DNA quantity evaluated with spectrophotometer, 260/230: Ratio of absorbance at 260 nm and 230 ng; 260/280: ratio of absorbance at 260 nm and 280 nm; µg Fluorometer: DNA quantity evaluated with fluorometer; QC Score: quality control score obtained by qPCR analyses representing the DNA integrity (a dimensionless number tending either to 1, in a context of highly conserved DNA, or to 0, in a scenario of diffuse DNA degradation).

| **ID patient** | **Cohort** | **Anatomical Site** | **ID sample** | **µg Spectrophotometer** | **260/230** | **260/280** | **µg Fluorometer** | **QC Score** |
| --- | --- | --- | --- | --- | --- | --- | --- | --- |
| 1 | A | Thyroid | 1 stdFFPE | 9.45 | 1.61 | 2.01 | 1.08 | 0.23 |
|  |  |  | 1 coldFFPE | 21.25 | 2.04 | 2.04 | 2.59 | 0.87 |
| 2 | A | Lung | 2 stdFFPE | 3.50 | 1.20 | 2.00 | 0.43 | 0.22 |
|  |  |  | 2 coldFFPE | 2.38 | 1.11 | 1.95 | 0.70 | 0.76 |
| 3 | A | Colon | 3 stdFFPE | 1.87 | 2.40 | 1.90 | 0.21 | 0.23 |
|  |  |  | 3 coldFFPE | 5.31 | 2.30 | 1.90 | 1.09 | 1.02 |
| 4 | A | Breast | 4 stdFFPE | 15.93 | 2.21 | 2.00 | 1.01 | 0.03 |
|  |  |  | 4 coldFFPE | 3.06 | 2.10 | 1.98 | 0.55 | 0.44 |
| 5 | A | Breast | 5 stdFFPE | 7.98 | 2.10 | 1.99 | 0.64 | 0.05 |
|  |  |  | 5 coldFFPE | 3.34 | 2.06 | 2.02 | 0.67 | 0.75 |
| 6 | A | Gastric | 6 stdFFPE | 2.35 | 1.86 | 1.90 | 0.52 | 0.15 |
|  |  |  | 6 coldFFPE | 3.05 | 1.90 | 2.30 | 0.34 | 1.01 |
| 7 | A | Breast | 7 stdFFPE | 9.30 | 1.76 | 1.88 | 1.12 | 0.29 |
|  |  |  | 7 coldFFPE | 3.05 | 1.80 | 1.98 | 0.40 | 0.41 |
| 8 | A | Colon | 8 stdFFPE | 10.50 | 1.90 | 1.94 | 0.77 | 0.07 |
|  |  |  | 8 coldFFPE | 42.00 | 2.20 | 1.99 | 9.00 | 0.69 |
| 9 | A | Colon | 9 stdFFPE | 5.44 | 1.80 | 2.00 | 0.84 | 0.15 |
|  |  |  | 9 coldFFPE | 35.46 | 2.00 | 1.90 | 16.38 | 0.63 |
| 10 | A | Lung | 10 stdFFPE | 11.28 | 1.88 | 1.90 | 0.94 | 0.13 |
|  |  |  | 10 coldFFPE | 30.00 | 2.00 | 1.98 | 5.10 | 0.46 |
| 11 | A | Lung | 11 stdFFPE | 17.94 | 1.98 | 1.96 | 2.90 | 0.13 |
|  |  |  | 11 coldFFPE | 34.50 | 2.10 | 1.90 | 8.76 | 0.81 |
| 12 | A | Thyroid | 12 stdFFPE | 2.26 | 1.70 | 1.93 | 0.90 | 0.14 |
|  |  |  | 12 coldFFPE | 3.76 | 2.20 | 2.00 | 0.85 | 0.24 |
| 13 | A | Gastric | 13 stdFFPE | 13.68 | 1.90 | 1.97 | 2.74 | 0.13 |
|  |  |  | 13 coldFFPE | 18.60 | 2.19 | 1.94 | 8.16 | 0.65 |
| 14 | A | Breast | 14 stdFFPE | 19.05 | 2.06 | 1.97 | 2.61 | 0.08 |
|  |  |  | 14 coldFFPE | 9.06 | 2.13 | 1.98 | 3.30 | 0.67 |
| 15 | A | Breast | 15 stdFFPE | 18.48 | 2.06 | 1.96 | 3.75 | 0.04 |
|  |  |  | 15 coldFFPE | 16.02 | 2.13 | 1.94 | 3.48 | 0.41 |
| 16 | A | Breast | 16 stdFFPE | 28.53 | 2.18 | 1.98 | 4.89 | 0.19 |
|  |  |  | 16 coldFFPE | 27.60 | 2.15 | 2.05 | 2.52 | 0.63 |
| 17 | A | Breast | 17 stdFFPE | 23.31 | 2.12 | 1.94 | 4.05 | 0.10 |
|  |  |  | 17 coldFFPE | 19.68 | 2.08 | 2.04 | 1.35 | 0.65 |
| 18 | A | Breast | 18 stdFFPE | 25.77 | 2.02 | 1.97 | 4.02 | 0.07 |
|  |  |  | 18 coldFFPE | 9.54 | 2.15 | 1.97 | 2.52 | 0.71 |
| 19 | A | Breast | 19 stdFFPE | 22.56 | 1.95 | 2.17 | 3.12 | 0.05 |
|  |  |  | 19 coldFFPE | 45.18 | 1.94 | 2.16 | 8.19 | 0.61 |
| 20 | A | Breast | 20 stdFFPE | 10.53 | 1.97 | 2.17 | 2.04 | 0.08 |
|  |  |  | 20 coldFFPE | 22.20 | 1.90 | 2.03 | 6.54 | 0.54 |
| 21 | A | Breast | 21 stdFFPE | 16.98 | 2.07 | 1.97 | 6.45 | 0.05 |
|  |  |  | 21 coldFFPE | 24.84 | 2.15 | 1.92 | 3.63 | 0.34 |
| 22 | B | Colon | 22 stdFFPE | 6.30 | 2.22 | 2.00 | 9.93 | 0.56 |
|  |  |  | 22 coldFFPE | 25.29 | 2.20 | 2.00 | 9.63 | 0.56 |
| 23 | B | Colon | 23 stdFFPE | 20.10 | 2.10 | 2.00 | 7.26 | 0.61 |
|  |  |  | 23 coldFFPE | 12.96 | 2.10 | 1.98 | 4.29 | 0.61 |
| 24 | B | Gallbladder | 24 stdFFPE | 16.35 | 2.10 | 1.95 | 6.87 | 0.59 |
|  |  |  | 24 coldFFPE | 31.44 | 2.20 | 2.00 | 11.79 | 0.82 |
| 25 | B | Colon | 25 stdFFPE | 15.18 | 2.20 | 2.00 | 5.31 | 0.67 |
|  |  |  | 25 coldFFPE | 28.95 | 2.20 | 2.00 | 9.30 | 0.89 |
| 26 | B | Colon | 26 stdFFPE | 19.59 | 1.85 | 1.99 | 5.46 | 0.55 |
|  |  |  | 26 coldFFPE | 24.87 | 2.22 | 2.00 | 7.29 | 0.69 |
| 27 | B | Colon | 27 stdFFPE | 15.88 | 2.10 | 2.02 | 6.30 | 0.64 |
|  |  |  | 27 coldFFPE | 22.05 | 1.99 | 1.98 | 10.71 | 0.64 |
| 28 | B | Lung | 28 stdFFPE | 22.11 | 2.01 | 2.20 | 9.39 | 0.62 |
|  |  |  | 28 coldFFPE | 18.21 | 2.01 | 2.20 | 9.01 | 0.73 |
| 29 | B | Colon | 29 stdFFPE | 22.05 | 2.20 | 1.99 | 7.12 | 0.80 |
|  |  |  | 29 coldFFPE | 27.72 | 2.20 | 2.00 | 9.39 | 0.80 |
| 30 | B | Spleen | 30 stdFFPE | 54.05 | 2.20 | 2.00 | 15.31 | 0.78 |
|  |  |  | 30 coldFFPE | 15.88 | 2.00 | 2.00 | 10.40 | 0.80 |
| 31 | B | Ovary | 31 stdFFPE | 16.70 | 1.98 | 2.01 | 9.01 | 0.66 |
|  |  |  | 31 coldFFPE | 20.48 | 2.02 | 1.98 | 6.30 | 0.70 |
| 32 | B | GIST | 32 stdFFPE | 13.99 | 1.99 | 2.05 | 7.23 | 0.80 |
|  |  |  | 32 coldFFPE | 27.63 | 2.06 | 2.13 | 5.15 | 1.02 |
| 33 | B | Liver | 33 stdFFPE | 27.57 | 2.03 | 2.11 | 15.64 | 0.86 |
|  |  |  | 33 coldFFPE | 55.87 | 1.97 | 2.15 | 8.68 | 0.98 |
| 34 | B | Colon | 34 stdFFPE | 7.06 | 2.06 | 1.44 | 2.08 | 0.61 |
|  |  |  | 34 coldFFPE | 10.83 | 2.01 | 1.66 | 1.25 | 0.74 |
| 35 | B | Lung | 35 stdFFPE | 26.65 | 2.09 | 2.14 | 3.37 | 0.63 |
|  |  |  | 35 coldFFPE | 20.19 | 2.06 | 2.03 | 5.45 | 0.63 |
| 36 | B | GIST | 36 stdFFPE | 3.36 | 1.70 | 2.00 | 0.22 | 0.68 |
|  |  |  | 36 coldFFPE | 4.56 | 1.90 | 2.00 | 0.30 | 0.76 |
| 37 | B | Bone | 37 stdFFPE | 18.12 | 2.18 | 1.89 | 3.45 | 0.58 |
|  |  |  | 37 coldFFPE | 22.98 | 2.20 | 1.92 | 4.51 | 0.58 |
| 38 | B | Colon | 38 stdFFPE | 33.48 | 2.10 | 1.99 | 3.33 | 0.73 |
|  |  |  | 38 coldFFPE | 24.24 | 2.10 | 2.04 | 3.79 | 1.04 |

**Supplementary Table 2: Details on DNA quantity and quality for 14 Cohort B paired samples after 6 months of storage included in the study.**

coldFFPE: cold formalin fixed paraffin embedded sample fixed; stdFFPE: standard (room temperature) formalin fixed paraffin embedded; µg Spectrophotometer: DNA quantity evaluated with spectrophotometer, 260/230: Ratio of absorbance at 260 nm and 230 ng; 260/280: ratio of absorbance at 260 nm and 280 nm; µg Fluorometer: DNA quantity evaluated with fluorometer; QC Score: quality control score obtained by qPCR analyses representing the DNA integrity (a dimensionless number tending either to 1, in a context of highly conserved DNA, or to 0, in a scenario of diffuse DNA degradation).

| **ID patient** | **Cohort** | **Anatomical Site** | **ID sample** | **µg Spectrophotometer** | **260/230** | **260/280** | **µg Fluorometer** | **QC Score** |
| --- | --- | --- | --- | --- | --- | --- | --- | --- |
| 22 | B | Colon | 22 stdFFPE_6 | 11.5 | 2 | 1.98 | 4.2 | 0.38 |
|  |  |  | 22 coldFFPE_6 | 10.85 | 1.99 | 1.96 | 1.9 | 0.55 |
| 23 | B | Colon | 23 stdFFPE_6 | 10.89 | 1.98 | 1.89 | 5.2 | 0.31 |
|  |  |  | 23 coldFFPE_6 | 14.2 | 2 | 1.91 | 7.2 | 0.52 |
| 24 | B | Gallbladder | 24 stdFFPE_6 | 13.9 | 2 | 2 | 11.2 | 0.41 |
|  |  |  | 24 coldFFPE_6 | 11.5 | 1.98 | 2 | 2.3 | 0.79 |
| 25 | B | Colon | 25 stdFFPE_6 | 9.05 | 1.97 | 2.01 | 4.5 | 0.47 |
|  |  |  | 25 coldFFPE_6 | 8.60 | 1.99 | 2.01 | 5.1 | 0.83 |
| 26 | B | Colon | 26 stdFFPE_6 | 5.05 | 1.82 | 2.03 | 3.6 | 0.40 |
|  |  |  | 26 coldFFPE_6 | 6.05 | 1.85 | 2 | 4.1 | 0.57 |
| 27 | B | Colon | 27 stdFFPE_6 | 11.3 | 1.99 | 1.82 | 6.8 | 0.36 |
|  |  |  | 27 coldFFPE_6 | 12.3 | 2 | 1.92 | 5.9 | 0.60 |
| 28 | B | Lung | 28 stdFFPE_6 | 15.2 | 2.01 | 1.99 | 9.2 | 0.46 |
|  |  |  | 28 coldFFPE_6 | 12.3 | 2.02 | 1.99 | 7.5 | 0.70 |
| 29 | B | Colon | 29 stdFFPE_6 | 16.6 | 1.96 | 2.03 | 9.2 | 0.39 |
|  |  |  | 29 coldFFPE_6 | 12.3 | 1.97 | 1.98 | 5.8 | 0.71 |
| 30 | B | Spleen | 30 stdFFPE_6 | 11.3 | 1.85 | 1.9 | 10.2 | 0.34 |
|  |  |  | 30 coldFFPE_6 | 15.2 | 1.89 | 1.91 | 11.1 | 0.72 |
| 31 | B | Ovary | 31 stdFFPE_6 | 14.2 | 1.99 | 1.97 | 8.9 | 0.59 |
|  |  |  | 31 coldFFPE_6 | 14.8 | 1.99 | 1.95 | 9.1 | 0.69 |
| 32 | B | GIST | 32 stdFFPE_6 | 19.6 | 2.01 | 1.96 | 6.5 | 0.60 |
|  |  |  | 32 coldFFPE_6 | 18.1 | 2.03 | 1.94 | 7.6 | 0.81 |
| 33 | B | Liver | 33 stdFFPE_6 | 13.2 | 1.99 | 1.93 | 12.2 | 0.47 |
|  |  |  | 33 coldFFPE_6 | 12.2 | 1.92 | 1.95 | 11.1 | 0.88 |
| 34 | B | Colon | 34 stdFFPE_6 | 11.2 | 1.96 | 1.97 | 4.1 | 0.43 |
|  |  |  | 34 coldFFPE_6 | 16.3 | 1.94 | 1.98 | 3.5 | 0.62 |
| 35 | B | Lung | 35 stdFFPE_6 | 14.2 | 1.99 | 1.99 | 5.5 | 0.46 |
|  |  |  | 35 coldFFPE_6 | 18.2 | 1.96 | 1.99 | 6.2 | 0.61 |
| 36 | B | GIST | 36 stdFFPE_6 | 7.2 | 1.95 | 2.01 | 0.56 | 0.38 |
|  |  |  | 36 coldFFPE_6 | 6.3 | 1.99 | 2 | 0.65 | 0.55 |
| 37 | B | Bone | 37 stdFFPE_6 | 8.6 | 2.01 | 1.98 | 3.5 | 0.31 |
|  |  |  | 37 coldFFPE_6 | 9.2 | 2 | 1.95 | 2.5 | 0.52 |
| 38 | B | Colon | 38 stdFFPE_6 | 7.3 | 1.96 | 1.99 | 8.2 | 0.41 |
|  |  |  | 38 coldFFPE_6 | 7.8 | 1.98 | 1.98 | 7.2 | 0.79 |
